# Use of the House-Tree-Person Projective Drawings and Parental Styles Inventory in the Global Psychological Evaluation of Transgender Youth Who Seek Healthcare at the Gender Identity Program

**DOI:** 10.3389/fpsyg.2019.02488

**Published:** 2019-11-12

**Authors:** Bianca Machado Borba Soll, Angelo Brandelli Costa, Anna Martha Vaitses Fontanari, Ítala Raymundo Chinazzo, Dhiordan Cardoso da Silva, Karine Schwarz, Maiko Abel Schneider, Cesar Augusto Nunes Bridi Filho, Claudia Garcia de Garcia, André Real, Silza Tramontina, Maria Inês Rodrigues Lobato

**Affiliations:** ^1^Programa de Identidade de Gênero, Hospital de Clínicas de Porto Alegre (HCPA), Universidade Federal do Rio Grande do Sul (UFRGS), Departamento de Psiquiatria e Medicina Legal, Porto Alegre, Brazil; ^2^Pontifícia Universidade Católica do Rio Grande do Sul (PUCRS), Departamento Programa de Pós Graduação em Psicologia, Porto Alegre, Brazil; ^3^Department of Psychiatry and Behavioral Neuroscience, McMaster University, Hamilton, ON, Canada

**Keywords:** psychological evaluation, parental styles, house-tree-person projective drawings, gender incongruence, gender dysphoria, transgender youth

## Abstract

The present study explores data collected in the psychological evaluation of transgender youth and their families who seek healthcare at the Gender Identity Program. Great psychosocial changes mark the transition from infancy to adulthood. Transgender youth may have these aspects of their developmental stage potentialized. A study was conducted with 23 transgender youth (mean age = 14 ± 2.38 years) and their caregivers. Eleven of the youngsters were assigned male at birth, while 12 were assigned female. The research protocol consisted of a survey and systematization of the data collected in the initial global psychological evaluation performed at the healthcare facility, including house-tree-person (HTP) projective drawings and the parental styles inventory. The present study aimed to explore the data collected during the psychological evaluation of youngsters diagnosed with gender incongruence, relating the HTP projective drawing technique to parental styles and gender trajectories. The results indicate two key points. One evidenced that parental styles could be either preventive or risk components in maintaining adequate socialization in these young people but not in affecting the level of gender dysphoria. The other was that coherence is introduced in the person’s perception of his or her projected self-image and his or her expressed gender as he/she becomes more comfortable in expressing his/her gender identity. Treating youngsters inherently brings ethical issues to clinical practice. Thus, global psychological evaluation tailored to this population is a fundamental resource that the psychology professional can use in consultations with youngsters because this tool brings a global understanding about the natural development cycle, facilitating the formulation of therapeutic conducts and exchanges within interdisciplinary transgender health care teams.

## Introduction

A transgender individual is a person whose gender identity does not correspond with their birth-assigned sex. The number of people with gender incongruence and/or gender dysphoria who seek assessment, support, and treatment at gender identity clinic services has increased over the years, requiring better qualification and standardization of care by all the professionals involved (Bouman et al., 2016). In Brazil, any transgender people diagnosed according to the ICD can undergo health services gender specifically, which is fully covered by the Brazilian Unified Health System (SUS). However, since a policy allowing hormonal therapy for transgender youth younger than 18 years is not available in Brazil, such treatment can be performed only within a research context.

### Youngsters and Gender Incongruence in Psychological Evaluation

Significant body, cognitive, and psychosocial changes mark the transition from infancy to adulthood. Transgender youth may have these aspects of their developmental stage potentialized if they have a poor peer social relations (De Vries et al., 2015). The bioecological development model (Bronfenbrenner, 1996) elucidates how interactions between immediate and distant environments of young people, such as families and their surrounding social context, can influence psychosocial aspects development. In addition, they often have difficulties accessing health services when they need. Psychological evaluation is a broad process in which the psychologist makes use of a variety of resources recognized by psychological science, such as tests and interviews, with defined clinical objectives for evaluation and interpretation of data (Cunha, 2000; Krug et al., 2016). This is a fundamental resource that the psychology professional can use in healthcare context with transgender youth because it brings global and embedded information that contributes to the formulation of therapeutic conduct and facilitates exchanges with interdisciplinary transgender health care teams. It is important to make sure that psychological evaluation is not used to interfere with the expressed gender. Its use is for evaluation of the global functioning, health demands, and guarantee of rights.

In psychology, puberty and adolescence are not necessarily synonymous. The former has a biological aspect and accompanies physiological changes toward sexual maturity; the latter refers to a phase marked by a psychosocial course from infancy to adulthood—with a cultural influence—in which the individual seeks in collective formations elements to establish his personal identity (Gleitman et al., 2003). In addition, concomitant with the maturational phases, it is important to understand the specificity of the gender trajectories that this transgender youth finds. Pinto and Moleiro (2015) developed a theoretical model of gender trajectories that shows the movement in five stages of development: (1) confusion and increasing sense of gender difference; (2) finding an explanation and a label: exploring identity; (3) deciding what to do and when: exploring options; (4) embracing gender identity: performing a new social identity and undergoing body modifications; and (5) identity consolidation and invisibility. Each stage reflects a specific form of gender identity management for a transgender person. Thus, psychological evaluation of transgender youth should consider the level of synchrony between emotional development and the stage of pubescent development (the relationship with the body and the self-image) together with the degree of social and familiar support. Above all, it is necessary to contextualize their stage of the gender trajectory, elucidate any internal and external resources, the strengths and the possible difficulties of their personal characteristics, and their surroundings that can impact the symptoms of gender dysphoria when present and its natural development cycle. From this perspective, the approach of the psychologist and the objective of the evaluation are therapeutic; therefore, they are not limited to the recommendation of a specific clinical treatment available or the confirmation or not of gender dysphoria. Some of the objectives include exploring gender identity, role, and expressions, addressing the negative impact of gender dysphoria and the social stigma attached to it, increasing social support, and improving the relationship with body image (Coleman et al., 2012). All of these conducts aim to alleviate the symptoms of gender dysphoria, when present, and to strengthen the autonomy of the youngsters in the face of possible alternatives of clinical treatment and ways to feel more comfortable expressing their gender identity.

In global assessments of development, it is common to use a standard test battery that includes quantitative and qualitative evaluation instruments. The use of the projective drawing test is one of the qualitative techniques used in the evaluation of children and adolescents. An important aspect that is sensitive to projective techniques is self-perception. Often youngsters are consolidating their gender expression, and this type of method can be a useful tool to help them. Previous studies have shown that youngsters with gender dysphoria tend to draw an individual with a sex opposite the one first assigned at birth (Zucker et al., 1983; Cohen-Kettenis and Pfäfflin, 2003; in these studies, the diagnostic classification was from the DSM-IV). There is an evolutionary pattern in the literature that points to a general tendency to draw a person with the same sex assigned at birth when asked to draw a person, but drawing the opposite sex assigned at birth had not been confirmed as an indicator of emotional difficulties (Arteche et al., 2010). However, considering the updating of nomenclatures, when studies depart from a perspective of gender identities instead of the assigned sex at birth, it is perceived that the drawing of the human figure indicates that subject and experienced gender are consistent and significant predictors of the gender of the drawn figure (Houston and Terwilliger, 1995). The drawing of the human figure has as its premise the projection of the image of the body itself in the drawing, that is, the conception that the subject has of his/her own body and his/her function in the social world (Buck, 2003). A study using a projective test on adults with gender dysphoria showed a tendency to subjectively perceive imperfection or personal inadequacy (Barišić et al., 2014). The same logic can be extended to young people. Compared to couples, transgender youth have lower self-perception, especially in physical self-worth, a fact that may be related to the high rates of co-occurring psychopathology (Alberse et al., 2019).

In addition to using different assessment techniques, the clinical management of gender questions in youngsters should include parental evaluation. The quality of the family functioning system is fundamental in the process of support and decision-making regarding the transition from infancy to adulthood of these transgender youth. Knowing the parental style helps to establish specific support strategies for caregivers according to their demands that are reflected in the overall well-being of the youngsters, strengthening the whole family as they experience this process. Therefore, parental style is defined as the set of parental attitudes used by caregivers to educate, socialize, and guide the behavior of their children (Gomide, 2006), encompassing everything that contributes to the emotional climate in which the child is educated (Darling and Steinberg, 1993). However, a review of the literature has revealed few studies with families of young transgender people necessitating greater exploration of this field of study (Dierckx et al., 2016).

The present study aimed to explore the data collected in the psychological evaluation of transgender youth seeking healthcare at the gender identity program relating the house-tree-person (HTP) projective drawing technique to parental styles and gender trajectories in order to better understand the different psychosocial facets that are involved in the therapeutic clinic of this population.

## Materials and Methods

### Participants

This study’s sample consisted of families seeking healthcare at the Gender Identity Program (PROTIG) at the Hospital de Clínicas de Porto Alegre (HCPA), located in southern Brazil. The sample consisted of 24 young people, aged 8–16 years, of whom 11 were assigned as male and 12 were assigned as female at birth, which meet criteria for gender incongruence, together with their caregivers (*n* = 34). The research protocol consisted of the survey and systematization of the data collected in the global psychological evaluation performed at the hospital. These data included different instruments and techniques, including structured interviews, cognitive tests, projective tests, morbidity evaluations, anamnesis, and clinical interviews. These were applied to each individual and to the caregiver who was present throughout the study. However, only instruments and techniques that were associated with the purpose of the article were used in this study. The exclusion criterion was a lack of a gender incongruence diagnosis (ICD-11) and a lack of a response from both parents to the parental styles inventory (PIS) (e.g., youngsters who were under state protection during the evaluation period).

### Technique

#### Psychological Clinical Interview

The psychological clinical interviews were the exploration of the first interviews conducted by a professional psychologist, allowing evaluation, intervention, and psychological support in the encounter between the young person and the therapist. They take into account the emotional development and the environmental factors that are involved in the life of the youngsters. Because the facility used in this study is a hospital healthcare system specializing in gender issues, it is common for a young person to enter his/her first psychotherapeutic interview with the expectation that the psychologist understands his/her questions about the incongruence between his/her experienced gender and assigned sex. From this meeting, it was possible to establish at which stage of the gender trajectory the participants were engaged. For this, the theoretical model of gender trajectories proposed by Pinto and Moleiro (2015) served as the basis for the collection of information. The interviews focused on the life histories, family, school experiences, community support, knowledge, and use of your legal rights and how the youngsters recognize and explore their gender identities.

### Instruments

#### Parental Styles Inventory

The parental styles inventory (PIS) (Gomide, 2006) is a validated in Brazil (Cronbach’s alpha values obtained showed internal consistency in all educational practices, ranging from 0.46 for maternal negative monitoring at 0.8663 for paternal moral behavior) (Sampaio and Gomide, 2017) and self-enforcing instrument, composed of seven categories of positive and negative parental educational practices. In this study, we considered the form of *paternal and maternal educational practices,* individually answered by each caregiver about the educational practices adopted with their children. The two positive educational practices are divided into (1) **positive monitoring**, which involves the proper use of attention and distribution of privileges, the proper establishment of rules, the continuous and safe distribution of affection, and the monitoring and supervision of school and leisure activities; (2) **moral behavior**, which implies promoting favorable conditions for the development of virtues, such as empathy, a sense of justice, responsibility, work, generosity, and knowledge of right and wrong about drug and alcohol use and safe sex, following the example set by the parents. The five negative educational practices are divided into (3) **Inconsistent punishment:** in which the parents are guided by their temperament at the time of punishing or reinforcing and not by the act practiced; (4) **Negligence**: a lack of attention and affection. (5) **Relaxed discipline**: the relaxation of established rules; (6) **Negative monitoring**: characterized by an excess of instructions regardless of their compliance and, consequently, by the generation of an atmosphere of hostile coexistence; (7) **Physical and psychological abuse**: characterized by discipline through negative corporal practices, threats, and blackmail of abandonment and humiliation of the child. A total of 42 items are presented, consisting of statements such as “I criticize anything my son does, how the room is messed up or his hair tousled,” and “I ask how your day was at school and listen carefully”. Caregivers should indicate how often they act in the situation described according to the legend: *Never* (if, on 10 occasions, he/she acted that way between 0 and 2 times); *Sometimes* (if, on 10 occasions, he/she acted that way 3–7 times); *Always* (if, on 10 occasions, he/she acted that way 8–10 times). The *parental style index* (*iep*) can range from −60 to +24. The result can be classified into four categories: (1) *Great Parental Style*: an absence of negative parenting practices; (2) *Regular Parental Style above average* and (3) *Regular Parental Style below average*: both include variations of positive and negative practices; and (4) *Risk Parental Style*: a predominant use of negative practices to the detriment of positive ones. Any outcome other than the Great Parenting Style demands orientation and interventions to different degrees in parental practices to avoid greater social and psychological harm to the youngster and his family.

#### House-Tree-Person Projective Drawing Technique

House-tree-person (Buck, 2003) is a validated instrument in Brazil population, approved by the Psychological Testing System (SATEPSI), in January 2004 (Conselho Federal de Psicologia, 2018), that uses the projective drawing technique of a house, a tree, and a person to obtain information about how the subject experiences his individuality in relation to others and to the environment around him. In this study, the application of HTP consisted of two phases. In the first, the individual was invited to make a drawing of a house, then of a tree, and finally of a person, one by one. The instruction was “I would like you to draw a **house**, I would like you to draw a **tree**, I would like you to draw a **person**”. Accordingly, participants were given white sheets of paper (21 by 29.7 cm) and a pencil. The second phase consisted of the *Inquiry*, in which structured questions are asked concerning the individual’s associations with aspects of each drawing. The interpretation protocol of this instrument has spaces for a notations of significant observations of the behavior that can complement the reported associations. A *List of Interpretive Concepts* provides a reference of common interpretive concepts so that they can be interpreted consistently. The clinical richness of HTP is in the possibility of stimulating projections of elements of different aspects of the *self* and of the interrelations of the individual with the environment inside the therapeutic system. Therefore, it allows degrees of subjectivity that enrich clinical evaluation. In the context of research, however, this instrument becomes more consistent when used in conjunction with other psychometric techniques, so that the elements found can be considered with in a global evaluation. Interpretation categories were positive when there were elements in the *List of Interpretive Concepts* consistent with the survey and were considered only when another etiological factor for the explanation was not found (e.g., psychiatric morbidity and cognitive aspects). We also chose only the indexes with significant distributions in the sample above 50%.

**Figure 1:**
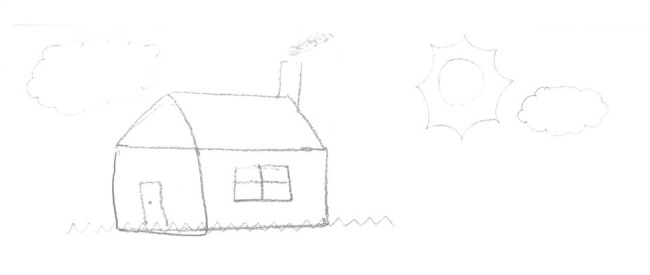
Drawing of a house in HTP.

Figure 1 shows an example of a small house design located in the first quadrant. The Inquiry evoked the following exchange:

Therapist: “What does this house need the most?” Why?

Answer: It needs people living inside of it so that it could be joyful and safe.

### Structured Interview

The structured interview used in the study was the same one used in the Mexican study (Robles et al., 2016) but translated, cultural adapted, and applied to the Brazilian population (Soll et al., 2018; Lobato et al., 2019). For this study, three categories were considered: diagnostic criteria, psychological distress, and loss in social functioning. The survey instrument, based on the participants’ reports, evaluated a particular period of time, i.e., when they first became consciously aware that they might be transgender (incongruence between one’s experienced gender and assigned sex). To fulfill the distress criteria, daily suffering (sadness and anxiety) must have been experienced for at least 6 continuous months. The intensity of emotion, as well as the intensity of psychological distress caused by the conflict between an incongruous assigned sex and gender identity, was assessed using a Likert scale varying from 1 (very little) to 5 (very strong).

### Procedure

Data collection was conducted between May 2014 and December 2018. Typically, transgender youth follow a particular route through the Brazilian Unified Health System (SUS), whereby they go through other health services (primary and secondary care) and screening before being referred to specialized service (tertiary care). The research was conducted at the Clinical Research Center of the Hospital de Clínicas de Porto Alegre (HCPA). At the first meeting, the psychological clinical interviews were carried out, lasting a maximum of three sessions. For application of the remaining protocol, two sessions were utilized with no more than 15 days between them. It is totalizing no more than five meetings. There were moments when only parents or only the children were interviewed based on the demands of the instruments. All psychological evaluation, clinical interview, and application of the instruments were performed by the same psychology professional. Trained and experienced in psychological testing and healthcare of youngsters.

### Ethics Statement

Researchers initially secured approval from the Ethics Committee of the Hospital de Clínicas de Porto Alegre (protocol no. 140274). All participants, both young people and their caregivers, were informed of the purposes of this research and then invited to participate. All subjects gave written informed consent.

### Data Analysis

Descriptive, statistical analysis of central tendency, and *Fisher exact test* was performed in SPSS version 21.0.

## Results

### Descriptive Analyses

A total of 23 young people, aged 8–16 years (mean age = 14 ± 2.38 years), were included, together with 34 their caregivers. Of these, 21 were mothers, aged 27–61 years (mean age = 43 ± 9 years), and 13 were fathers, aged 30–64 years (mean age = 45.8 ± 9.5 years). The demographic characteristics of the caregivers are shown in Table 1. Exclusion criterion was a lack of a response from both parents to the parental styles inventory (PIS).

**Table 1:** Demographic characteristics of the caregivers.

	Mother *n* = 21	Father *n* = 13	Total *N* = 34
**Formal study**			
Uncompleted elementary school Only completed elementary school Only completed secondary school Completed higher education	3 (14.3) 3 (14.3) 8 (42.9) 7 (33.3)	— 4 (30.8) 4 (30.8) 5 (38.4)	3 (8.8) 7 (20.6) 12 (35.3) 12 (35.3)
**Employment status**			
Employed Not employed	14 (66.7) 7 (33.3)	12 (92.3) 1 (7.7)	26 (74.3) 8 (23.5)

A majority of the transgender youth had studied in public school (60.9%) and 34.7% (8) had reported taking a leave of absence from their studies for reasons related to the incongruence between their assigned sex and gender identity.

### Parental Styles Inventory Results

Table 2 describes the results from the parental styles inventory.

**Table 2:** Parental styles inventory (PIS) results.

	Mother parental styles % (*n*)	Father parental styles % (*n*)	Total *N* = 34
Great parental style	38.1 (8)	7.7 (1)	9
Regular parental style above average	4.8 (1)	15.4 (2)	3
Regular parental style below average	28.6 (6)	30.8 (4)	10
Risk parental style	28.6 (6)	46.2 (6)	12

### House-Tree-Person Projective Drawing Technique Results

Only four indexes described by the List of Interpretive Concepts (Buck, 2003) were found that were significantly distributed (above 50%) in this sample: *Social withdrawal* (66.7%); A*ttachment security* (70.8%); *Insecurity with self-image* (87.5%); and *Feeling of physical inadequacy* (75%).

Only two subjects drew a figure of the same sex as the one assigned to them at birth. These two subjects were in the first stages of their gender trajectories. Table 3 shows a summary of the stages of gender trajectory for the participants. All other participants drew the figure based on their experienced gender. None of the participants was in the last stage of the gender trajectory.

**Table 3:** Stages of the gender trajectory.

	Transgender youth % (*n*)	Mean age (years)	Minimum–maximum (years)
Confusion and increasing sense of gender difference	17.4 (4)	11.25	9–13
Finding an explanation and a label: exploring identity	26.0 (6)	15.16	13–16
Deciding what to do and when: exploring options	39.1 (9)	14.4	8–16
Embracing gender identity	17.4 (4)	14.0	11–16
Identity consolidation and invisibility	—	—	—

**Figure 2:**
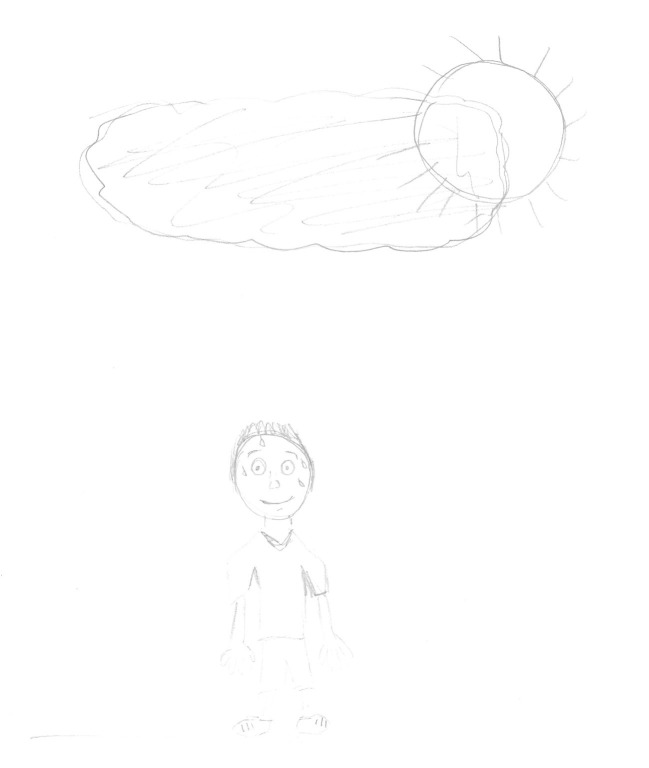
Drawing of person in HTP. Inquiry: Outside it is always very hot, and so it is common to sweat a little.

Figure 2 shows an example of a drawing made by a young person assigned female at birth and whose gender identity is male. The stage of his gender trajectory was e*mbracing gender identity.* The drawing was made in the third quadrant with central tendency (the sheet has been rotated in the figure below).

### Diagnostic Criteria and Psychological Distress Related to Gender Incongruity

All participants (*n* = 23) said they felt significant psychological distress (sadness and anxiety) related to their gender identity from the moment they realized the incongruity. Moreover, 78.2% (*n* = 18) of the participants reported a strong or very strong level of distress. All participants had confirmation of the diagnosis of gender incongruence (ICD-11) and gender dysphoria (DSM-5) after the evaluation and had indication of follow-up at the Gender Identity Program.

### Associations Between Parental Style and House-Tree-Person Positive Indexes, Psychological Distress Related to Gender Incongruity and Loss in Social Functioning

The Table 4 shows the Associations between Parental Style and HTP Positive Indexes, Psychological Distress Related to Gender Incongruity and Loss in Social Functioning. The *Fisher exact test* showed that there was only an association between *Mother Parental Styles* and *Absence from Their Studies* (*p* < 0.05).

**Table 4:** Associations between parental style and HTP positive indexes, psychological distress related to gender incongruity and loss in social functioning.

	Mother parental styles		Father parental styles	
	*N* (%)	p^**^	*N* (%)	p^**^
Social withdrawal	15 (71.4)	0.574	10 (76.9)	0.238
Attachment security	15 (71.4)	0.884	8 (61.5)	0.361
Insecurity with self-image	18 (85.7)	1.000	11 (84.6)	0.244
Feeling of physical inadequacy	15 (71.4)	0.574	9 (69.2)	0.099
Distress related to gender incongruity	21 (100)	0.101	13 (100)	0.179
Absence from their studies	8 (38.1)	0.028^*^	3 (23.1)	1.000

* *Correlation is significant at the 0.5 level*;

** *Fisher exact test*.

## Discussion

To understand the behavior and socialization of children and adolescents, we must consider the parental styles they receive from their family nucleus. The results of this study show that most caregivers (62% of mothers and 94.4% of fathers) use negative parental strategies in caring for their sons. It was also evident that parental styles could be either preventive or risk components in maintaining an adequate socialization for these transgender youth but not in affecting the level of gender dysphoria. The only significant association found in this sample was that of mother parental styles with absence from their studies. This means that the better the quality of the maternal parenting style, more chance the youngsters stays in their studies. Another study done in the Brazilian transgender persons seeking health services gender specifically also had a similar indicator. Most people with gender incongruence who reported taking a leave of absence from their work stated that it was due to social stigma and prejudice (Soll et al., 2018). This is important because it indicates that the impact on the impairment of social functions may be more related to social context than to dysphoria. This does not eliminate the intensity of the psychological distress caused by the conflict between the perception of a body being incongruent and current gender identity; it only suggests that the more social support individuals have, the less of an impact that distress will have on their social lives.

Other research involving general population samples show associations between the quality of parental caregiving and child development, psychosocial functioning, and school performance (Baumrind, 1991; Véronneau and Dishion, 2010). A coherent hypothesis is that socialization undoubtedly begins in family relationships but expands and correlates in different social systems. Thus, individuals are affected by their surrounding environmental factors at different levels of the social system, as suggested by the Bioecological Model of Development (Bronfenbrenner, 1996). Therefore, the impact of social stigma on dysphoria symptoms may come from more external strata of society, as the result of social withdrawal and attachment security were significant in the sample and did not indicate association with parental styles. In any case, the results of a lower prevalence of *Great Parental Style* are a strong indicator of the importance of therapeutic and psychoeducational interventions with caregivers to promote the care and functionality of these families.

The results show that the phase of the gender trajectory is quite significant and is not directly related to age or pubescent phase but rather to strengthening personal gender identity. In the sample studied, only those who were in the first stage, *confusion and an increasing sense of gender difference,* drew the figure according to the sex assigned at birth. A possible hypothesis for this finding is that as the stages of the gender trajectories evolve and that the concept of living and expressing himself or herself in the way that he or she identifies is a real possibility, a coherence is introduced in the person’s perception of his projected self-image and his expressed gender. Even so, the projective test also pointed to a perception of physical inadequacy and insecurity with self-image. This result is expected since self-esteem and satisfaction with body image, in addition to dysphoria, can be severely disrupted in transgender youth by rigid binary normative standards. The search for gender affirmation treatments is an attempt to resolve the dissonance between self-image, the internal model of self-perception, and the body. Strengthening personal identity in the context of the transition from childhood to adulthood is a challenge of different proportions for transgender youth. The current social environment reacts in a negative way to spontaneous expressions that do not correspond to the representations of enforced gender roles. The concept of a “safe enough holding environmental” (Winnicott, 1983) is associated with educational and clinical practices that are useful in current discussions about management with transgender youth. The safe enough holding environmental necessarily presupposes the consideration of the singularity, needs, demands, desires, resources, and rhythms of each young person. Any attempt to transform singular adaptation in a general way harms the whole set of developments and interactions with the social environment. Thus, the therapeutic environment is expected to be able to accommodate these young people without reproducing the normative social stereotypes. For this, it is assumed, before the technique or approach used, to think about the identity of gender and its expressions on a collective and not an individual perspective, which happens with all people and not only with transgender individuals.

## Conclusion

Complementing projective and psychometric techniques is a useful tool in the psychological clinical management of gender questions in youngsters; projective tests can detect aspects of the dynamics and personality or raise research hypotheses with their results that would be difficult to access by psychometric tests. Specifically, the HTP Projective Drawing Technique tends to be less invasive and follows a validated methodology that accesses emotional contents of perception and interaction with the environment that would often take much longer to be detected and analyzed in therapeutic processes. In addition, it is a way of valuing the individuality with which each subject lives and perceives their condition. Due to the importance of understanding the youth in their family and social contexts, extending the assessment not only to parents and family but also to school and other social dimensions are ways to elucidate the impact of the environment on the individual’s life.

However, resolving this incongruence between self-image and gender patterns may have some complementary fronts, such as clinical interventions at the somatic level and in the progressive deconstruction of binary social stereotypes at the therapeutic and social level.

## Limitations

The present study has some methodological limitations. First, the sample size was limited. Second, in spite of the clinical enrichment of the projective practices, they do not allow quantitative measurements, which are important for research purposes. In a future study, other psychometric scales can be used to measure self-image and the relationship with the body in conjunction with the projective test. In addition, the individuality and peculiarity of each child and adolescent who undergoes a psychological evaluation should always be prioritized. This study analyzes the average of a group and cannot be used to characterize an individual within the group.

## Data Availability Statement

All datasets generated for this study are included in the article/supplementary material.

## Ethics Statement

The studies involving human participants were reviewed and approved by. Researchers initially secured approval from the Ethics Committee of the Hospital de Clínicas de Porto Alegre (protocol no. 140274). Written informed consent to participate in this study was provided by the participants’ legal guardian/next of kin.

## Author Contributions

BS, AB, and ML conceived of the presented idea. BS performed the research protocol. ST helped and supervised the project. ÍC, DS, CB, AR, CG, MS, AV, and KS contributed to the interpretation of the results. All authors discussed the results and contributed to the final manuscript.

### Conflict of Interest

The authors declare that the research was conducted in the absence of any commercial or financial relationships that could be construed as a potential conflict of interest.
